# Laser-treated wood for high-efficiency solar thermal steam generation†

**DOI:** 10.1039/d2ra02918a

**Published:** 2022-08-31

**Authors:** Shu-Wei Wang, Han-Lin Xie, You-Yi Xia, He-Xin Zhang, Keun-Byoung Yoon

**Affiliations:** School of Textile and Material Engineering, Dalian Polytechnic University Dalian China; School of Chemistry & Chemical Engineering, Anhui University of Technology China; Department of Polymer Science and Engineering, Kyungpook National University South Korea

## Abstract

Solar-driven water vaporization is considered one of the most sustainable ways to solve water scarcity. The design of highly efficient solar absorber systems has received extensive attention. Here, we report a novel light absorption material for water evaporation using laser-treated wood. The obtained laser-treated wood possesses interconnected 3D porous networks formed by the random construction of carbon arrays and a hydrophilic surface due to the oxygen implantation by laser treatment. When under 1 sun solar-simulated light irradiation (1 kW m^−2^), the surface temperatures of dry and water-saturated wood reach 59.5 °C and 40.4 °C, respectively, indicating good heat localization. As a result, the laser-treated wood under 1 sun illumination shows high solar to vapor efficiencies of 93.1% and 92.6% for pure water and seawater, respectively, which are higher than that of most wood-based reported photo-thermal conversion materials. Therefore, the fabricated laser-treated wood may pave the way for harvesting solar energy to produce clean water at low cost.

## Abstract 2

Solar-driven water vaporization is considered one of the most sustainable ways to solve waterscarcity.

## Graphical abstract

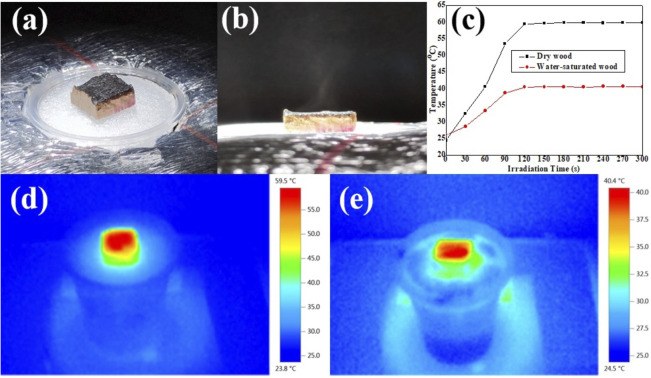

## Introduction

Fresh water is recognized as a global strategic resource that is critical to maintaining human living standards as well as economic development and social progress.^1–3^ Although water is one of the most abundant materials on Earth, with about 70% of the Earth's surface being covered by water, water from the oceans and seas is too salty to drink directly. Simultaneously, in recent decades, the booming population and industrial growth have resulted in serious pollution, causing a severe shortage of drinkable water, which has become an urgent issue to be solved.^4,5^ Therefore, various kinds of techniques have been reported, including solar to vapor generation, thermal distillation, membrane filtration, photo-catalysis and electrodialysis.^6–12^ Among them, solar to vapor generation using sunlight as the energy source is emerging as a promising and eco-friendly technique to solve the problems of water contamination and shortage.^13–18^ Solar energy is by far the most exploitable, renewable, and sustainable resource. According to the literature, the energy delivered by the sun to the earth in 1 hour is more than all of the energy consumed by humans in an entire year.^19–23^ Thus, in recent years, solar steam generation has attracted a great deal of attention in clean water production. In a typical solar distillation system, the solar evaporator is the key part, which captures solar energy, converts it to heat, and thereafter drives water evaporation from all kinds of water sources (such as seawater and polluted water).^24–29^ Various photo-thermal conversion materials with extremely high light absorption and diverse heat management strategies have been developed to achieve solar steam generation in the past few years, leading to a state-of-the-art solar to vapor generation efficiency higher than 90%. For example, Li *et al.* reported a mass-produced carbon nanosheet framework, which exhibited a solar to vapor efficiency of 93% with a solar water evaporation rate of ∼1.4 kg m^−2^ h^−1^. Yang *et al.* fabricated a hydrogel-based solar steam generator that exhibits a maximum solar thermal efficiency of 91.5% with an evaporation rate of 1.4 kg m^−2^ h^−1^. Yu *et al.* reported a light-absorbing hydrogel with highly hydratable polymer networks. The evaporation rate of 1 sun could be increased up to 3.6 kg m^−2^ h^−1^ at an energy efficiency of ∼92%.^30–32^

Currently, solar to vapor generation based on biomaterials, such as wood and mushrooms, holds great promise in alleviating fresh water crises. For example, wood exhibits many advantages for application in solar to vapor generation devices, such as good hydrophilicity, lightweight structure, inherent microchannels for water transport, and excellent thermal insulation. Hu *et al.* reported a self-regenerating solar to vapor evaporator based on a rationally designed channel-array structure for long-term antifouling solar desalination. Their solar evaporator exhibits an energy efficiency of 75% in 20 wt% NaCl. Guo *et al.* reported alkali-activated 3D carbon foams as solar evaporation devices based on natural wood. A solar-to-thermal conversion efficiency of 80.1% was achieved.^33,34^ Thus, wood is considered as an attractive raw material for water distillation devices. *Paulownia* is a genus of fast-growing tree species under natural and cultivated conditions that is native to China.^36^ Due to their rapid growth and value in the timber market, many *Paulownia* species are cultivated in several temperate zones around the world, including China, Japan, America and Australia.^37–39^ Besides, paulownia wood has low density (∼0.26 g cm^−3^), high heat insulation properties, is ring porous, straight-grained and mostly knot-free with a large pore size. Thus, paulownia wood is one of the ideal biomaterials for application in solar to vapor generation devices.

According to the literature, laser-induced graphene (LIG) has been successfully formed on wood surfaces under ambient conditions without the need for inert atmospheres or chemical vapor deposition furnaces.^35^ LIG on wood surfaces can be easily and simply patterned into various shapes and depths through computer software design. Therefore, in this study, the surface of paulownia wood was treated by laser to form a 3D carbon layer. The resultant carbon layer could act as a solar absorber and the untreated wood substrate acts as the water transport of the solar to vapor generation system. The effects of treatment conditions on the structure and performance of the wood were investigated. On the basis of this research, the rate of 1 sun solar vapor generation could be increased to up to 2.283 kg m^−2^ h^−1^ at an energy efficiency of 93.1%.

## Experimental

### Material preparation

Dried paulownia wood was chosen as the substrate and cut into 2 cm × 2 cm blocks (∼5 mm thick). The resultant paulownia wood blocks were treated by laser under air exposure. Laser induction was conducted on a DIAOTU (K6) laser platform with a maximum laser power of 3000 mW. The device has 3 parameters to set: contrast ratio (0–253), power percentage (0–100%) and depth (0–100). In this study, the contrast ratio, power percentage and depth were fixed at 120, 60% and 20, respectively.

### Material characterization

The morphologies of the natural and treated paulownia wood were characterized by scanning electron microscopy (SEM) using a JEOL JSM 6490-LV. The temperature of the solar to vapor generator was monitored using an IR camera (TESTO 868). The contact angles of the samples were measured using a DSA-100 optical contact angle meter (Kruss Company Ltd., Germany) using liquid droplets of water.

### Solar to vapor generation experiments

The water evaporation performance experiments were conducted using a homemade solar to vapor generation testing system. A solar simulator (PLS-SXE300) with a 300 W Xenon lamp (Perfectlight Scientific Pty Ltd., Beijing, China) was used as a light source and the solar intensity was adjusted using a Newport optical power meter (model: 1830-C). Wood with a thickness of ∼5 mm was fixed on a polystyrene foam holder and the foam was bridged over the beaker. The mass of the water loss was measured by a lab balance with a 0.1 mg resolution and calibrated to weights heavier than the total weight of the setup. All evaporation rates were measured after stabilization under 1 sun for 30 min.

## Results and discussion

**Fig. 1:**
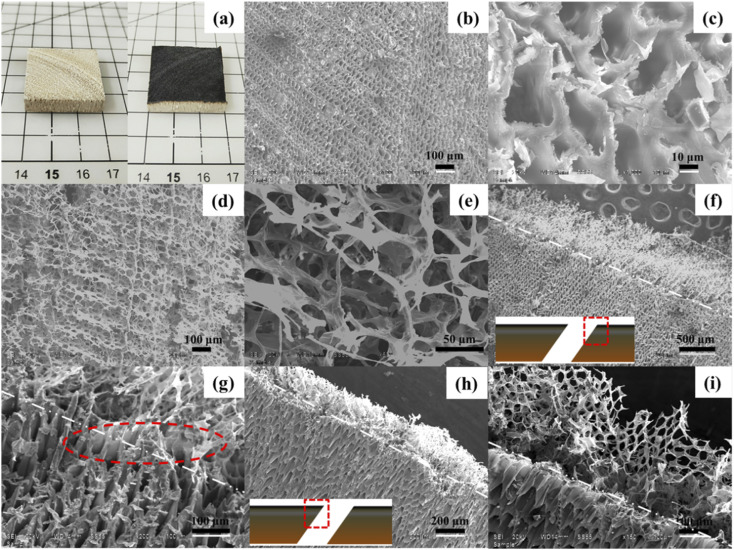
Morphologies of wood and treated wood. (a) Photographs of wood and laser-treated wood; (b and c) SEM images of wood at different magnification; (d and e) SEM images of the laser-treated wood surface at different magnification; (f and g) SEM images of the oblique section (obtuse angle) at different magnification; (h and i) SEM images of the oblique section (sharp angle) at different magnification.

Fig. 1(a) shows the paulownia wood before and after treatment with a laser. It was found that the color of the wood surface changed from light yellow to black due to the high temperature of laser treatment. The structure of wood was further characterized by SEM. As shown in Fig. 1(b) and (c), the cross-section of natural wood exhibited a typical pore structure (vessel of wood) with a diameter of 40 μm. The vessel of wood could continuously supply water by capillary action for solar to vapor generation. Fig. 1(d) and (e) show the front surface of laser-treated wood. The vessel of wood was completely destroyed and a vertical black carbon array layer was formed. The distance between two formed carbon layers was in the range of 50–100 μm, which is larger than the vessel diameter of wood. Fig. 1(f–i) show the oblique section of treated wood at different magnification. As can be seen from Fig. 1(f–i), the generated carbon grows like grass on the surface of the wood and the interface between carbon and wood can be easily distinguished. In addition, it can be seen from the marked part of Fig. 1(g) that the produced carbon is directly connected to the vessel wall of the wood. The thickness of the carbon array layer was evaluated; the thickness was in the range of 200 to 400 μm. Fig. 1(i) shows that the resultant carbon is a honeycomb sheet-like structure and vertical to the surface of the wood. The diameter of the pore on the carbon sheet was around 40 μm.

**Fig. 2:**
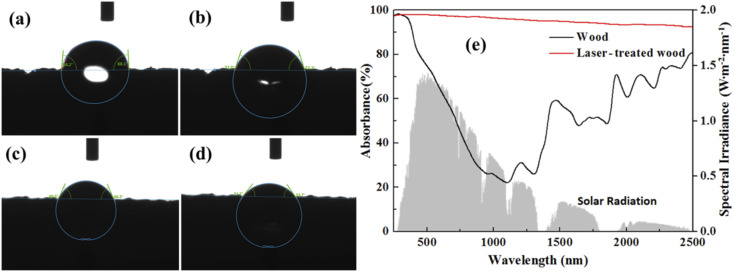
Contact angle of laser-treated wood at (a) 1 s and (b) 2 s; contact angle of wood at (c) 1 s and (d) 2 s; (e) UV-vis spectra of natural wood and laser-treated wood.

The water contact angles were measured by using a contact angle meter to characterize the wettabilities of the wood and laser-treated wood surfaces. As shown in Fig. 2(a) and (b), when the water droplet is placed on the carbon array of laser-treated wood, the contact angle is 88.1° at 1 s, and then quickly changes to 74.4° at 2 s. Meanwhile, with natural wood (Fig. 2(c) and (d)), the contact angle is 66.5° at 1 s and 53.7° at 2 s. Finally, the water totally disappears at 5 s. These results indicate that natural wood is more hydrophilic than the laser-treated wood, which favors the infiltration and transport of water. The relatively low hydrophilicity of laser-treated wood may be due to the reduced polar group content of wood during the laser treatment process. In terms of optical performance, the untreated wood exhibits low absorbance in the visible-NIR region (500–1500 nm), in which the solar spectrum (air mass 1.5 global, AM 1.5 G) has high intensity (Fig. 2(e)). Interestingly, as shown in Fig. 2(e), the formed carbon layer on the wood surface exhibits a broad and strong absorbance (>90%) from 280 to 2500 nm, which is significantly higher than that of natural wood. This fact should be attributed to the strong photo-absorption of the carbon layer as well as the increased surface area of wood, which results in the multi-scattering and absorption of the incident light. By taking advantage of the good absorption properties, the resultant 3D carbon-based materials can achieve higher light-absorbing capacity than others.

**Fig. 3:**
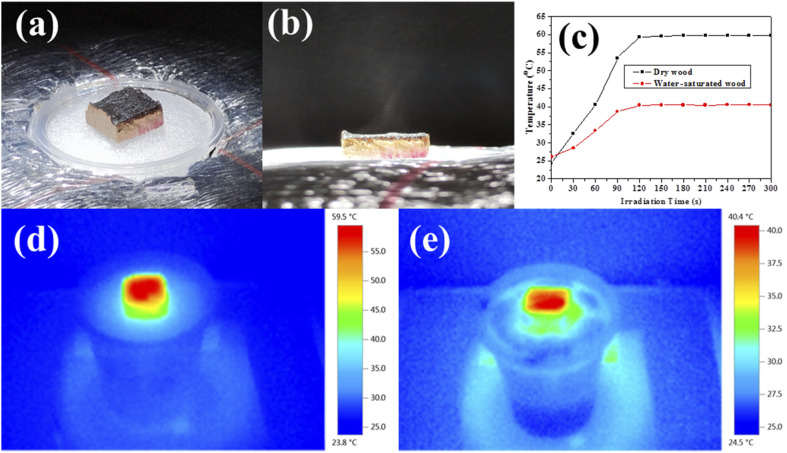
(a and b) Digital images of the solar to water generator; (c) temperature change curves of wood and water-saturated wood under 1 sun illumination; infrared images of (d) dry and (e) water-saturated laser-treated wood under 1 sun illumination.

**Fig. 4:**
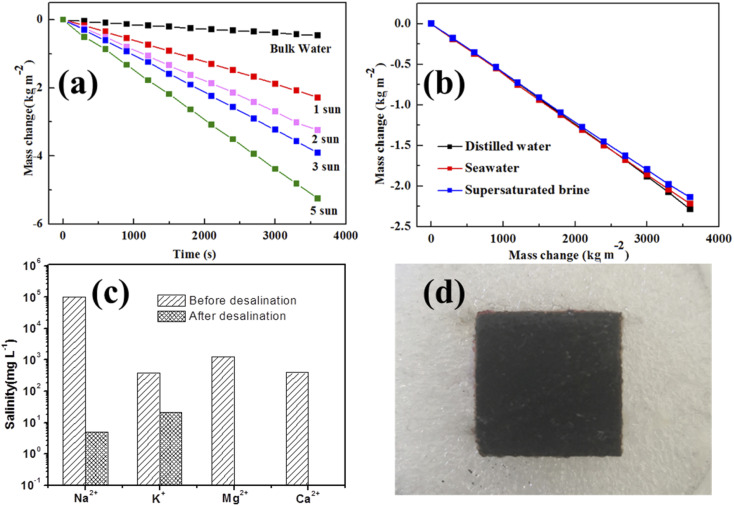
The steam generation performances of laser-treated wood under different solar illuminations. (a) The curves of water mass change *vs.* time under 1, 2, 3 and 5 sun illumination. (b) The curves of seawater and supersaturated brine mass change *vs.* time under 1 sun illumination. (c) Ion concentration of seawater (from Xinghai Square, Dalian, China) before and after desalination. (d) The surface of laser-treated wood after 10 h of continuous desalination.

It is well known that a broad and strong photo-absorption confers high photo-thermal effects.^40^ The photo-thermal performances of the resultant laser-treated wood were investigated under irradiation with a solar simulator (1.0 kW m^−2^). For dry laser-treated wood, its temperature goes up rapidly from room temperature (24.4 °C) to 59.5 °C (Fig. 3(a)). When the wood is saturated with water, its average surface temperature increases slowly from 26.1 °C (initial water temperature) to 40.4 °C after 120 s under the same irradiation (Fig. 3(b)). The solar to vapor generation properties were investigated and the results are given in Fig. 4. The whole solar to vapor generator was placed on a precision balance and exposed to an illumination intensity adjustable solar simulator. The water evaporation rates were calculated by recording the mass change as a function of time under different solar intensities. Each experiment was repeated 3 times, and the results were calculated from the average value of 3 results. According to the experimental results, the maximum error of each experimental data does not exceed 5%. According to recent studies, eqn (1) was used to calculate the energy conversion efficiency and evaluate the overall solar to vapor efficiency of the samples.1
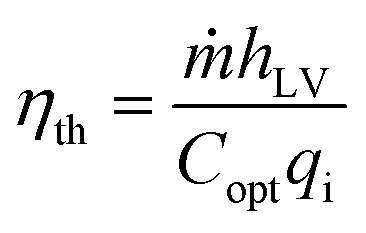
where *ṁ* is the mass flux, *h* is the heat of phase change (*h* = 2260 kJ kg^−1^), *C* is the optical concentration, *q* is the nominal direct solar irradiation of 1 kW m^−2^, and *η* is the energy conversion efficiency.^41–43^

At a 1 kW m^−2^ solar intensity, the mass losses for wood and laser-treated wood are measured to be 2.283 kg m^−2^ h^−1^ and 0.682 kg m^−2^ h^−1^, respectively, while it is 0.132 kg m^−2^ h^−1^ for pure water without a solar absorber. As the solar illumination intensity increases, the evaporation rate of laser-treated wood increases accordingly. On the basis of eqn (1), the energy conversion efficiencies of the wood and laser-treated wood were calculated to be 93.1% and 27.8% at 1 kW m^−2^, respectively. However, the water evaporation efficiency of bulk water without a solar absorber is only 0.458 kg m^−2^ h^−1^ at 1 kW m^−2^. The better evaporation efficiency of laser-treated wood could be attributed to the high light absorption, excellent heat localization and unimpeded water transport. The effects of solar illumination intensity on the water evaporation rates were also investigated. The weight losses of water evaporation with laser-treated wood at the different light densities of 1, 2, 3 and 5 kW m^−2^ were 2.283, 3.242, 3.904 and 5.251 kg m^−2^ h^−1^, respectively.

In order to investigate the practical applicability of laser-treated wood under extreme conditions, the time-dependent steam generation rate of the evaporator in practical seawater and supersaturated NaCl solution was studied. Fig. 4(a) and (b) shows the mass changes of water, practical seawater and supersaturated NaCl solution under different solar illumination intensities. It was found that the mass changes of practical seawater and supersaturated NaCl solution are slightly lower than that of pure water. This could be attributed to the partially blocked transport pathway of water. However, the laser-treated wood demonstrated stable steam generation with no visible salt deposition even with seawater under 1 kW m^−2^ irradiation for 10 h (Fig. 4(d)). The quality of the collected water for drinking was further investigated by measuring the concentrations of the elements in it. Fig. 4(c) compares the concentrations of primary elements (Na, K, Mg and Ca) in seawater before and after desalination. The concentrations of Na, K, Mg and Ca in seawater are 100.9 g L^−1^, 374 mg L^−1^, 1259.5 mg L^−1^, and 395.4 mg L^−1^, respectively, while after desalination, the concentration of the primary ions in seawater was obviously reduced, and the concentrations of Na, K, Mg and Ca reduced to 5.08 mg L^−1^, 20.07 mg L^−1^, 0 mg L^−1^ and 0 mg L^−1^, respectively, indicating that the collected water meets the standard of drinking water quality (WHO). Thus, this work provides a simple and effective way to produce drinkable water through the steam generation of seawater or other sources of water with laser-treated wood.

## Conclusions

To summarize, in this study, we fabricated a highly efficient wood-based solar absorber through a simple laser treatment process. The resultant laser-treated wood possesses an interconnected 3D porous network formed by the random construction of carbon arrays. The carbon layer on the wood surface exhibits a broad and strong absorbance (>90%) from 280 to 2500 nm, which is significantly higher than that of natural wood. Thus, the highest solar to vapor efficiency of laser-treated wood is 93.1% under 1 sun illumination. With regards to the practical applicability of laser-treated wood for solar to vapor generation, the concentration of primary ions in seawater was obviously reduced and the collected water meets the standard of drinking water quality (WHO).

## Conflicts of interest

There are no conflicts to declare.

## Supplementary Material

RA-012-D2RA02918A-s001

RA-012-D2RA02918A-s002
