# Supplementary material for: Laser-treated wood for high-efficiency solar thermal steam generation
Source: RSC Adv. 2022 Aug 31;12(38):24861–7. doi: 10.1039/d2ra02918a (PMC9428656; doi:10.1039/d2ra02918a)
Supplement: RA-012-D2RA02918A-s002 [file RA-012-D2RA02918A-s002.pdf]

Electronic Supplementary Material (ESI) for RSC Advances.
This journal is © The Royal Society of Chemistry 2022

Laser Treated Wood for High Efficiency Solar Thermal Steam Generation

Shu-Wei Wang[1], Han-Lin Xie[2], You-Yi Xia[2*], He-Xin Zhang[2*], Keun-Byoung Yoon[3*]

[1]School of Textile and Material Engineering, Dalian Polytechnic University, Dalian, China.

[2]School of Chemistry & Chemical Engineering, Anhui University of Technology, China

[3]Department of Polymer Science and Engineering, Kyungpook National University, South Korea

Table S1. A comparison of the wood based solar-thermal steam generation system from the literatures.

Wood	Method	Solar intensity (kW/m^2)	Mass change (kg/m^2·h)	Efficiency (%)	Ref.
Balsa wood	Assemble of carbon dots within wood microchannel	1	2.27	92.5	1
Basswood	Nanoparticles deposited on wood surface	10	11.8	85	2
Basswood	Carbonized wood with drilled channel-array	1	1.04	75.1	3
Nature wood	Drop casting of GO on the wood surface	1	1.64	91.8	4
Poplar/Pine/ Cocobolo	Carbonized wood	1	< 1	< 90	5
Balsa wood	CNT-modified flexible wood membrane	10	11.22	81	6
Wood	Flame treated wood	1	-	72	7
Cunninghamia lanceolata	Carbonized wood	1	1.45	91.3	8
Basswood	Spray-coated with graphite	1	1.2	80	9
Natural Wood	Carbonized wood	1/10	1.08/12.26	74/89	10
Natural Wood	Alkali treatment	1	1.26	80.1	11

Paulownia wood	Laser treated wood surface	1	2.28	93.1	This work
Pines	Laser treated wood surface	1	1.97	80.3	
Toona sinensis (A. Juss.) Roem	Laser treated wood surface	1	2.01	82.0	
Ziziphus jujuba Mill	Laser treated wood surface	1	1.67	68.1	

1 Hou Q, Xue C R, Li N*, et al.* Self-assembly carbon dots for powerful solar water evaporation. Carbon, 2019, 149: 556-563

2 Zhu M, Li Y, Chen F*, et al.* Plasmonic Wood for High-Efficiency Solar Steam Generation. Advanced Energy Materials, 2018, 8: 1701028

3 Kuang Y, Chen C, He S*, et al.* A High-Performance Self-Regenerating Solar Evaporator for Continuous Water Desalination. Adv Mater, 2019, 31: 1900498

4 Kim K, Yu S, An C*, et al.* Mesoporous Three-Dimensional Graphene Networks for Highly Efficient Solar Desalination under 1 sun Illumination. Acs Appl Mater Inter, 2018, 10: 15602-15608

5 Jia C, Li Y, Yang Z*, et al.* Rich Mesostructures Derived from Natural Woods for Solar Steam Generation. Joule, 2017, 1: 588-599

6 Chen C J, Li Y J, Song J W*, et al.* Highly Flexible and Efficient Solar Steam Generation Device. Adv Mater, 2017, 29: 1701756

7 Xue G, Liu K, Chen Q*, et al.* Robust and Low-Cost Flame-Treated Wood for High-Performance Solar Steam Generation. Acs Appl Mater Inter, 2017, 9: 15052-15057

8 Peng-Fei L, Lei M, Ziyang D*, et al.* A mimetic transpiration system for record high conversion efficiency in solar steam generator under one-sun. Materials Today Energy, 2018, 8: 166-173

9 Li T, Liu H, Zhao X*, et al.* Scalable and Highly Efficient Mesoporous Wood-Based Solar Steam Generation Device: Localized Heat, Rapid Water Transport. Adv Funct Mater, 2018, 28: 1707134

10 Liu H, Chen C, Chen G*, et al.* High-Performance Solar Steam Device with Layered Channels: Artificial Tree with a Reversed Design. Advanced Energy Materials, 2018, 8: 1701616

11 Qiu P, Liu F, Xu C*, et al.* Porous three-dimensional carbon foams with interconnected microchannels for high-efficiency solar-to-vapor conversion and desalination. J Mater Chem A, 2019, 7: 13036-13042
